# Convergent structural brain alterations in chronic pain: a multi-metric individual participant data meta-analysis

**DOI:** 10.1093/braincomms/fcag146

**Published:** 2026-04-24

**Authors:** Ryan W J Loke, Oscar Ortiz, Sylvia M Gustin, Michèle Hubli, Clas Linnman, Abigail Livny, Yann Quidé, Paulina S Scheuren, John L K Kramer

**Affiliations:** Department of Anesthesiology, Pharmacology, and Therapeutics, Faculty of Medicine, University of British Columbia, Vancouver, Bc v6t 1z3, Canada; International Collaborations on Repair Discoveries (ICORD), University of British Columbia, Vancouver, Bc v5z 1n1, Canada; International Collaborations on Repair Discoveries (ICORD), University of British Columbia, Vancouver, Bc v5z 1n1, Canada; School of Biomedical Engineering, Faculty of Applied Sciences, University of British Columbia, Vancouver, Bc v6t 2b9, Canada; NeuroRecovery Research Hub, School of Psychology, The University of New South Wales (UNSW) Sydney, Sydney, NSW 2052, Australia; Centre for Pain IMPACT, Neuroscience Research Australia, Randwick, NSW 2031, Australia; Spinal Cord Injury Center, Balgrist University Hospital, University of Zurich, Zurich 8008, Switzerland; Neuroscience Center Zurich, ETH Zurich and University of Zurich, Zurich 8057, Switzerland; Department of Psychiatry, Massachusetts General Brigham & Harvard Medical School, Boston, MA 02115, USA; Clinical Brain Imaging R&D Center, Sheba Medical Center, Tel Aviv 5262000, Israel; Sagol School of Neuroscience, Faculty of Medical and Health Sciences, Tel Aviv University, Tel Aviv 6997801, Israel; NeuroRecovery Research Hub, School of Psychology, The University of New South Wales (UNSW) Sydney, Sydney, NSW 2052, Australia; Centre for Pain IMPACT, Neuroscience Research Australia, Randwick, NSW 2031, Australia; Department of Anesthesiology, Pharmacology, and Therapeutics, Faculty of Medicine, University of British Columbia, Vancouver, Bc v6t 1z3, Canada; International Collaborations on Repair Discoveries (ICORD), University of British Columbia, Vancouver, Bc v5z 1n1, Canada; Department of Anesthesiology, Pharmacology, and Therapeutics, Faculty of Medicine, University of British Columbia, Vancouver, Bc v6t 1z3, Canada; International Collaborations on Repair Discoveries (ICORD), University of British Columbia, Vancouver, Bc v5z 1n1, Canada

**Keywords:** chronic pain, brain morphology, individual participant data meta-analysis, curvature

## Abstract

Chronic pain is a leading contributor to all-cause morbidity and disability, encompassing numerous biopsychosocial dimensions that persistently engage complex networks of brain regions. Meta-analyses have advanced our understanding of structural brain differences in chronic pain but rely exclusively on summary statistics which may introduce heterogeneity related to completeness of reporting and differences in methodological approaches. To address these limitations, we conducted the first individual participant data (IPD) meta-analysis of brain structure alterations in chronic pain. Using traditional morphometric measures (i.e. volume, cortical thickness, and surface area) and differential-geometric shape metrics (i.e. intrinsic and extrinsic curvature), we aimed to reveal alterations in brain structure convergent across chronic pain conditions. We hypothesized that chronic pain would be associated with region-specific grey matter reductions in regions previously implicated in chronic pain (e.g. parahippocampal gyrus and insula) and explored whether curvature metrics would reveal additional structural changes. Anatomical MRI images from eight publicly available datasets spanning five conditions and 401 individuals with chronic pain (and 245 age- and sex- matched healthy controls) were analysed: (i) knee osteoarthritis, (ii) chronic low back pain, (iii) fibromyalgia, (iv) migraine, and (v) primary trigeminal neuralgia. FreeSurfer was used to parcellate T1-weighted anatomical images, and metrics for cortical and subcortical regions were extracted. Meta-analysis revealed a range of structural changes in the brain associated with chronic pain. Cortical thinning and volume loss were small and localized to the temporo-occipital regions, including bilateral volumetric reductions in the entorhinal cortex in individuals with chronic pain. Increases in intrinsic curvature were widespread, involving 49 out of 68 cortical regions. No significant alterations were detected in subcortical volumes. Intrinsic curvature and subcortical volumetric estimates had higher levels of inter-study heterogeneity compared to other metrics, reflecting potential condition and sample-specific variability. Leveraging harmonized processing across a large sample size, our novel IPD meta-analysis highlights both widespread and region-specific structural remodelling of chronic pain-related neuroanatomy.

## Teaser

Loke *et al*. investigated neuroanatomical alterations across multiple chronic pain conditions. They found that chronic pain is linked to region-specific reductions in grey matter volume and cortical thickness, alongside global increases in intrinsic curvature. These findings support the view of chronic pain as a disorder of large-scale cortical reorganization.

## Graphical Abstract

**Graphical Abstract:**
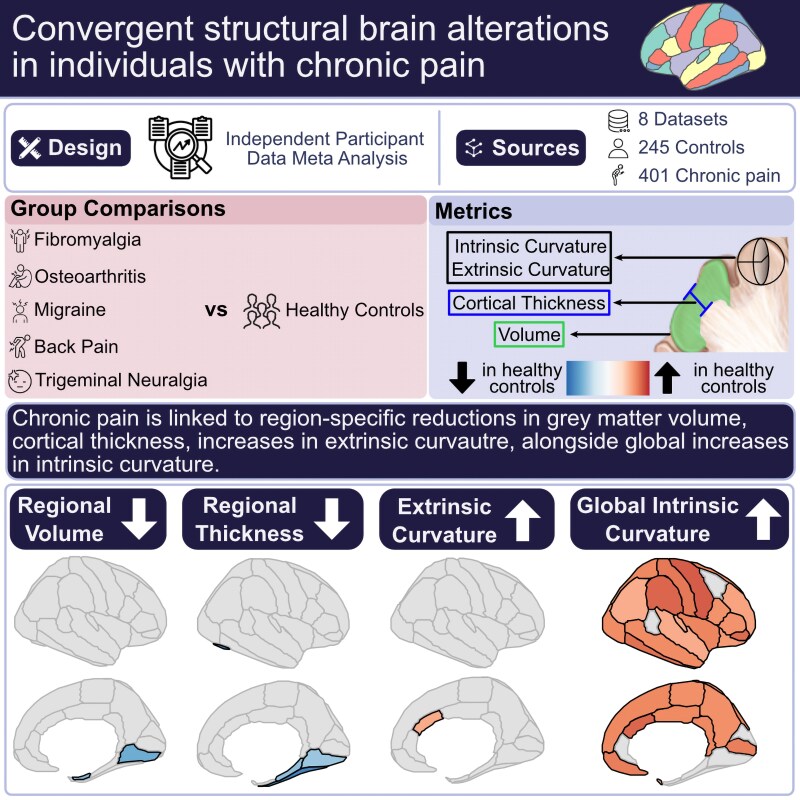

## Introduction

Chronic pain is a leading contributor to all-cause morbidity and disability.^1^ It encompasses biopsychosocial dimensions, including sensory, emotional, and behavioural components, that persistently engage a complex network of brain regions.^2,3^ A comparison of brain structure in individuals with chronic pain to healthy sex- and age- matched controls has revealed clear alterations in brain anatomy. This has been done on a study-by-study basis, revealing marked differences in cortical structure^4-6^ and subcortical volumes,^7,8^ and then synthesized in conventional, aggregate meta-analyses.^9,10^ Reported changes include decreased grey matter volume in several cortical areas like the cingulate cortex^8,9,11,12^ and the parahippocampal gyrus.^8,9^ Volumetric decreases were also observed in subcortical regions like the amygdala^8^ and thalamus.^7,11,13^ While a valuable tool in evidence-based research, conventional meta-analyses rely exclusively on published summary statistics. This introduces biases related to the completeness of reporting and methodological approach taken by the original investigators.^14,15^ Furthermore, heterogeneity across meta-analytic findings, such as reported decreases^7,13^ and increases^7^ in regional volumes (e.g. thalamus), may reflect condition-specific changes.

Chronic pain is increasingly understood as a disorder of distributed brain networks rather than a purely peripheral phenomenon.^16^ Persistent nociceptive input and ongoing affective and cognitive engagement with pain are thought to drive experience-dependent neuroplasticity in regions involved in sensory processing, salience detection, and cognitive control, such as the insula, cingulate cortex, and limbic structures.^17-19^ Structural brain metrics such as cortical thickness, surface area, volume, and curvature are sensitive to long-term changes in brain morphology and cortical folding, providing indirect markers of chronic alterations in neural organization associated with sustained pain processing.^18,20^

Individual participant data (IPD) meta-analyses are now widely embraced as a ‘gold standard’ for meta-analyses.^21^ By processing raw T1-weighted magnetic resonance imaging (MRI) scans with a uniform pipeline, IPD meta-analyses reduce variability attributed to methodological heterogeneity (e.g. FreeSurfer versus FSL) and also support subgroup analyses (e.g. sex).^14^ With access to raw data, IPD meta-analyses enable examination of both conventional morphometric metrics—grey matter volume (total amount of tissue), cortical thickness (distance between pial and white matter surfaces), and surface area (total cortical expanse)—and differential-geometric measures that are rarely available in published summaries. These include extrinsic (mean) curvature, which quantifies how the cortical surface folds in three-dimensional space (reflecting local gyrification), with increased extrinsic curvature reflecting increased folding complexity.^22^ Intrinsic (Gaussian) curvature captures the fundamental geometry of the surface itself: whether regions are dome or saddle shaped, independent of how the structure folds. At the millimetre scale, intrinsic curvature is thought to reflect differential cortical expansion and local connectivity organization, potentially capturing subtle microarchitectural reorganization beyond conventional metrics.^23^ Compared to mega-analyses—which use IPD in a single pooled statistical model—IPD meta-analyses typically produce highly similar results.^24-26^ Where IPD meta-analyses excel is the transparent study-level estimates and heterogeneity statistics, making cross-condition differences clear, prompting further investigation into certain subgroups. The main challenge facing IPD analyses is access to IPD, which is often hampered by data sharing concerns. Despite the abundance of IPD in public repositories like OpenNeuro,^27^ an IPD meta-analysis examining changes in brain structure across chronic pain conditions is currently lacking.

Although peripheral mechanisms and clinical phenotypes vary across different chronic pain conditions, the subjective burden of persistent pain shows cross-diagnostic similarity.^28^ Neuroimaging studies across diagnoses consistently implicate common brain regions and networks, supporting the hypothesis that chronic pain may be associated with convergent neuroanatomical alterations that transcend diagnostic boundaries.^11,29^ We therefore adopt a cross-condition IPD meta-analysis strategy, utilizing a single, harmonized segmentation pipeline, and pooling across conditions to increase power, report condition-specific effects, and detect convergent neuroanatomical differences. In this study, we analysed structural MRI data from eight publicly available data sets across five chronic pain conditions: knee osteoarthritis (OA), fibromyalgia (FM), chronic low back pain (CLBP), primary trigeminal neuralgia (PTN), and migraine. We examined a range of structural brain metrics—grey matter volume, cortical thickness, surface area, and both intrinsic and extrinsic curvature—in cortical regions and subcortical volumes parcellated using FreeSurfer. Taking an IPD meta-analysis approach, we hypothesized that chronic pain is associated with morphological brain changes consistent with prior voxel-based meta-analyses, including localized reductions in regions like the parahippocampal gyrus^10^ and insula.^10,30^ Given the absence of prior meta-analytic work on differential-geometric shape measures in chronic pain, we took an exploratory approach to curvature-based metrics to assess whether they capture structural reorganization beyond what is detectable with conventional metrics.

## Materials and methods

**Figure 1:**
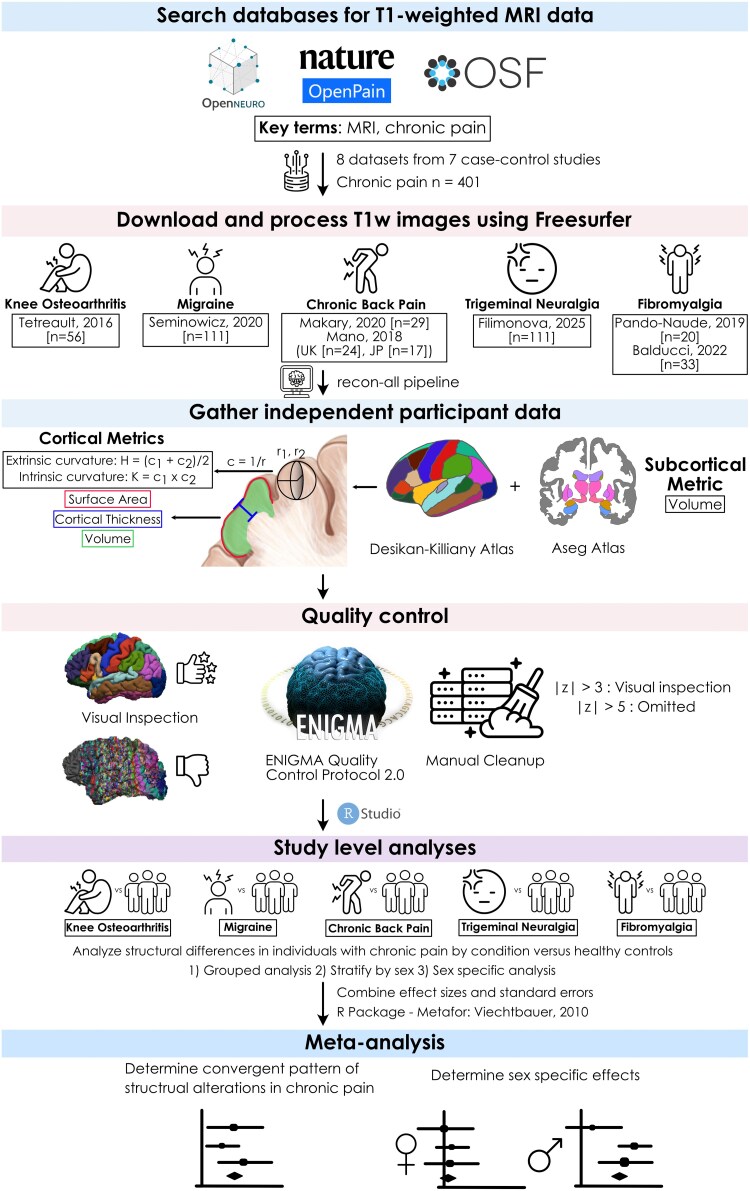
**Independent participant data meta-analysis pipeline.** The process includes database searching, processing of images, data extraction, quality control, study-level analysis, and meta-analysis. Abbreviations: OSF, Open Science Framework; UK, United Kingdom; JP, Japan; T1W, T1-weighted; ENIGMA, Enhancing Neuroimaging Genetics through Meta-analysis.

A summary of the overall workflow is shown in Fig. 1.

### Search strategy

We performed a search on OpenNeuro^27^ and the Open Science Framework^31^ databases using the search terms ‘MRI’ and ‘chronic pain’. We further queried ‘Scientific Data’ using the same search terms and examined study folders in the ‘OpenPain Project’ database. Finally, a manual search of published studies that captured anatomical MRI data in a sample of individuals with chronic pain was performed to determine additional sources of data (e.g. GitHub and Figshare). We limited the addition of data from studies that included anatomical data from age- and sex-matched healthy controls and were published to ensure quality and provenance.

### MRI preprocessing—cortical parcellation

Three-dimensional T1-weighted (3D T1w) anatomical MRI images from each participant were downloaded and processed locally using the recon-all function from FreeSurfer (Version 7.4.1; https://surfer.nmr.mgh.harvard.edu/, Harvard University, Boston, MA, USA).^32^ Processing was performed on Windows 11 x64 using Windows Subsystem for Linux (Ubuntu 22.04). This involved automated cortical and subcortical parcellation, including motion correction, skull stripping, and intensity normalization to improve image quality. In brief, the brain image is inflated into a spherical image for alignment to the Talairach atlas coordinates, and FreeSurfer then applies advanced segmentation to identify brain regions and cortical structures. Surface-based morphometry then parcellates each brain into 68 cortical regions using the Desikan–Killiany atlas. The estimated total intracranial volume (ICV) was extracted from the Talairach registration transform for use in volumetric adjustments.

From each parcellated region, eight structural metrics were extracted, and five were analysed in this study—surface area (SurfArea), grey matter volume (GrayVol), cortical thickness (ThickAvg), mean curvature (MeanCurv, extrinsic curvature), and Gaussian curvature (GausCurv, intrinsic curvature). The remaining three (NumVert, FoldInd, CurvInd) were excluded due to limited biological relevance.

### Subcortical segmentation

Recon-all also segments subcortical structures and calculates their volumes, using FreeSurfer’s registration of the anatomical image to the probabilistic Aseg atlas of structure location and shape.^33^

The subcortical structures analysed were the thalamus, accumbens, caudate, putamen, pallidum, hippocampus, and amygdala. Volumes for biologically relevant reference compartments including the brain stem, ventral diencephalon, corpus callosum (anterior, mid-anterior, central, mid-posterior, posterior), cerebellar cortex, cerebellar white matter, and the ventricular system (lateral, third, fourth, and inferior lateral) were also included. We excluded regions that are primarily technical (white matter/non-white matter hypointensities), vascular or ependymal (left/right vessel, choroid plexus), the optic chiasm, and aggregate measures (e.g. total cerebrospinal fluid), due to their lack of interpretability as independent regions of interest in our study.

### Quality control

One participant who did not report sex was included in all primary group-level analyses but was excluded from sex-stratified analyses. Another participant was excluded for failure to meet chronic pain inclusion criteria, as original study documentation indicated subacute rather than chronic pain. Following these exclusions, a total of 401 individuals with chronic pain and 245 healthy controls were included in data analyses.

All cortical measurements and subcortical volumes were standardized ($Z_{\mathrm{score}}=\frac{x-\mu }{\sigma }$) for each region and metric across individuals. Here, *x* denotes the raw value for an individual, *μ* is the sample mean, and *σ* is the population standard deviation. Data points with |*z*| > 3 (conventional threshold for extreme outlier) were flagged for visual inspection, and data points with |*z*| > 5 were auto-excluded as implausible outliers. Visual inspection was done using the Enhancing NeuroImaging Genetics Through Meta-Analysis Consortium (ENIGMA, https://enigma.ini.usc.edu/) Cortical Quality Control Protocol (Version 2.0).^34^ This generates standardized slice-wise visualizations of cortical and subcortical segmentations to facilitate the identification of errors. Measurements were retained if visual inspection confirmed anatomically plausible segmentation consistent with the Desikan–Killiany atlas; otherwise, they were excluded. In total, 56 of 219 640 data points were excluded during quality control, leaving 219 584 data points for analysis.

### Data analysis

Statistical analyses were performed using R Version 4.5.1 on Windows 11 x64. Each cohort was compared to their respective age- and sex-matched healthy controls for each region and metric. For volumetric measurements (cortical and subcortical volumes), regional volumes were adjusted for total ICV at the study level prior to effect size estimation. Specifically, within each study, regional volumes were residualized with respect to ICV using linear regression, and the resulting residuals were used for all subsequent group comparisons and effect size calculations. Parametric and non-parametric tests were used according to the results of normality tests (Shapiro–Wilk test). Effect sizes (Hedges’ *g*) were calculated using the R package esvis (Version 0.3.1)^35^ due to the smaller sample sizes of certain studies. The effect size formula is $g=\left(\frac{M_{1}-M_{2}}{SD_{\mathrm{pooled}}}\right)J$, where $M_{1}-M_{2}$ is the difference in means (i.e. healthy controls − chronic pain). The small sample bias correction factor *J* is calculated by $J=1-\frac{3}{4*\mathrm{df}-1}$ with df (degrees of freedom) = $\;n_{1}+n_{2}-2$ where $n_{1}$ and $n_{2}$ denote the respective sample sizes of each group. Standard error for each effect size was calculated using the formula $SE_{g}=\surd \left(\frac{n_{1}+n_{2}}{n_{1}*n_{2}}+\left(\frac{g^{2}}{2*(n_{1}+n_{2})}\right)\right)$. Confidence intervals were calculated using the following formulas, $CI_{\mathrm{lower}}=g-1.96*SE_{g}$ and $CI_{\mathrm{upper}}=g+1.96*SE_{g}$. The Benjamini–Hochberg method was used to correct for false discovery rate (FDR) with a significance level set to α=0.05. *P*-values were adjusted within each metric-specific family (e.g. thickness of cortical regions).

### Meta-analysis

The meta-analysis was conducted using the package metafor (Version 4.8-0).^36^ Effect sizes and variances were modelled with rma() using a random-effects with a restricted maximum likelihood (REML) estimator, chosen for its favourable statistical properties and reduced bias in small samples. Heterogeneity was assessed using $I^{2}$, $\tau^{2}$, and Cochrane’s Q and associated *P*-value for each of the 340 region-metric combinations and 32 subcortical volumes. Cochrane’s Q *P*-value was corrected for multiple comparisons using the Benjamini–Hochberg method, with a significance level set to α=0.05. Prediction intervals were also calculated per region-metric using the package meta (Version 8.1-0).^37^

### Sex effects

Datasets were stratified by sex, and analyses were rerun within cohorts from each study. Hedges’ *g* effect sizes and their respective variances were computed separately for males and females within each study, region, and structural metric. Sex-specific effect size estimates were compared using a two-sided Wald *Z* test of difference for each region-metric combination. For each male and female effect size estimate per region-metric combination, the formula $z=\frac{g_{m}-g_{f}}{\surd ({\mathrm{SE}}_{m}^{2}+{\mathrm{SE}}_{f}^{2})}$ was used to calculate a Wald *z*-score. Significance was assessed at α=0.05 corresponding to |*z*| > 1.96. To correct for multiple comparisons, each *Z* value was converted into a two-sided *P*-value using the following formula: p=2[1−Φ(|z|)], where *z* is the Wald-type statistic and Φ(z) is the cumulative distribution function (Φ(x)=P(Z≥x)) of the standard normal distribution. The cumulative distribution function returns the probability that a standard normal variable is greater than or equal to *x*. The returned *P*-value is then corrected for multiple comparisons using the Benjamini–Hochberg method, with a significance level set to α=0.05.

## Results

### Dataset breakdown

Eight datasets comprising T1w MRI scans from seven previously published case–control studies were included in this study. These datasets span five chronic pain conditions: knee OA,^38^ CLBP,^39,40^ FM,^41,42^ migraine,^43^ and PTN.^44^ Each dataset included age- and sex- matched healthy controls. Demographic details and scan acquisition parameters are provided per study in Table 1. Methodological details for each dataset are available in the original publications cited, and individual study-level comparative statistics are provided in the supplementary table.

**Table 1:** Demographic details and MRI acquisition parameters per data set

	Tétreault *et al*., 2016^38^	Makary *et al*., 2020^39^	Mano *et al*., 2018^40^	Mano *et al*., 2018^40^	Pando-Naude *et al*., 2019^41^	Balducci *et al*., 2022^42^	Seminowicz *et al*., 2020^43^	Filimonova *et al*., 2025^44^
Condition	Osteoarthritis	Chronic low back pain	Chronic low back pain	Chronic low back pain	Fibromyalgia	Fibromyalgia	Migraine	Primary trigeminal neuralgia
**Demographic details**								
Location	Illinois, USA	Connecticut, USA	Cambridge, UK	Osaka, Japan	Mexico City, Mexico	Queretaro, Mexico	Baltimore, USA	Novosibirsk, Russia
HC N (F/M)	20 (10/10)	33 (14/19)	39 (14/25)	17 (11/6)	20 (20/0)	33 (33/0)	35 (30/4)	48 (27/21)
HC mean age (F/M)	57.9 (58/57.8)	31.2 (31.1/31.3)	39.1 (42.6/37.2)	44.4 (47/39.7)	42.1 (42.1/NA)	41.5 (41.5/NA)	37.4 (38.2/32)	56.2 (54.8/58)
HC SD age (F/M)	6.7 (3.13/9.15)	9.6 (8.59/10.6)	13.5 (13.9/13.2)	11.8 (8.72/16)	12.5 (12.5/NA)	6.03 (6.03/NA)	13.1 (13.1/13.8)	7.4 (6.24/8.42)
Pain N (F/M)	56 (30/26)	29 (17/12)	24 (12/12)	17 (12/5)	20 (20/0)	33 (33/0)	111 (97/14)	111 (65/46)
Pain mean age (F/M)	57.9 (58.2/57.6)	30.8 (33.4/27.2)	46.3 (49/43.5)	44 (46.2/38.8)	46.4 (46.4/NA)	41.7 (41.7/NA)	36.8 (36.3/40.8)	59 (60.4/57)
Pain SD age (F/M)	7.0 (7.59/6.29)	11.6 (12.6/9.32)	11.3 (9.48/12.6)	11.4 (9.44/14.9)	12.4 (12.4/NA)	6.09 (6.09/NA)	12.3 (12.3/11.8)	12.3 (12.6/11.7)
Duration of pain (years)	11.2	5.5	11.6	10.4	5.3	8.1	NA	7
Medications	Average medication quantification scale = 7.5	NSAID (no info) Cyclobenzaprine 6% Tramadol 3% No opioids	NA	NA	NA	57% use medications daily no opioids, full details available	No opioids, 15% used	Carbamazepine 95% Gabapentin 9% Amitriptyline 7% Pregabalin 5% Duloxetine 2% Lamotrigine 1% Oxcarbazepine 1% Dose available
Scan parameters								
Data acquired from	OpenNeuro	OpenPain	OpenPain	OpenPain	OpenNeuro	OpenNeuro	OpenNeuro	OpenNeuro
Sequence	MPRAGE T1w	MPRAGE T1w	MPRAGE T1w	MPRAGE T1w	FSPGR BRAVO	FFE SENSE T1w	MPRAGE T1w	(NA) T1w
TR/TE (ms)	2500/3.36	1900/2.52	2300/2.98	2250/3.06	7.7/3.2	7/3.5	2300/2.98	6.56/2.95
Flip (°)	9	9	9	9	12	8	NA	8
Voxel (mm)	1 × 1 × 1	NA	NA	NA	NA	1 × 1 × 1	1 × 1 × 1	NA
Matrix/FOV (mm)	256 × 256/256	256 × 256/NA	256 × 256/256	256 × 256/256	256 × 256/256	240 × 240/240	NA	256 × 256/256
Slices	160	176	176	208	168	180	NA	NA

NA, not available; HC, healthy controls; T1w, T1-weighted; MPRAGE, magnetization-prepared rapid gradient echo; FSPGR, fast spoiled gradient echo; BRAVO, brain volume imaging; FFE, fast field echo; SENSE, sensitivity encoding; TR, repetition time; TE, echo time; FOV, field of view.

### IPD meta-analysis results

**Figure 2:**
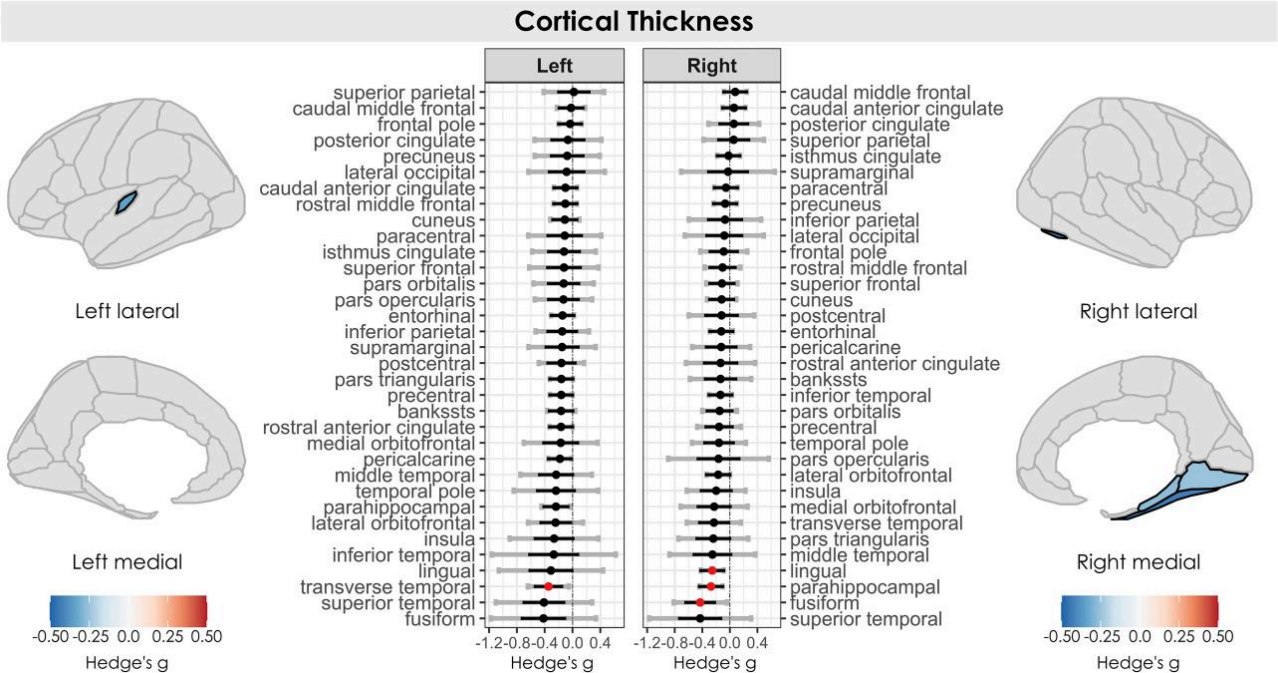
**Regional cortical thickness in individuals with chronic pain versus healthy controls.** Estimated effect sizes (Hedges’ *g*) for cortical thickness per region across *k* = 8 datasets, comparing individuals with chronic pain (*n* = 401) to healthy controls (*n* = 245), coloured by significance (red dot/black outline/coloured in = FDR *P* < 0.05). Positive effect sizes (red) indicate larger values in individuals with chronic pain, and negative effect sizes (blue) indicate smaller values in individuals with chronic pain. Black error bars indicate 95% confidence intervals, and grey error bars indicate prediction intervals.

Significant reductions in cortical thickness were observed in individuals with chronic pain (Fig. 2). Cortical thinning occurred in the right lingual gyrus, right parahippocampal gyrus, right fusiform gyrus, and left transverse temporal gyrus.

**Figure 3:**
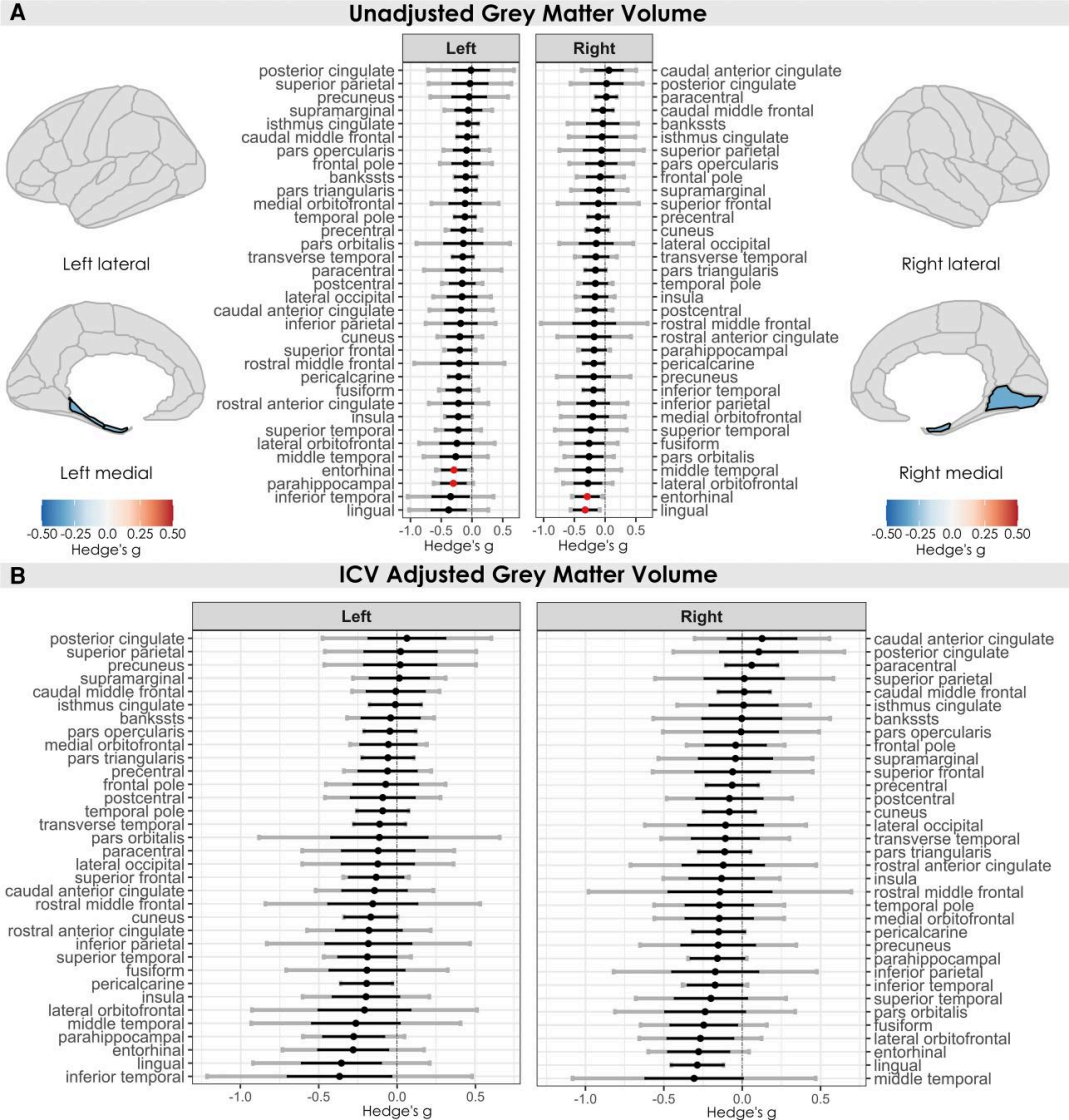
**Regional differences in grey matter volume in individuals with chronic pain versus healthy controls.** Estimated effect sizes (Hedges’ *g*) for volume per region across *k* = 8 studies, between individuals with chronic pain (*n* = 401) and healthy controls (*n* = 245). (**A**) Unadjusted estimated effect sizes (Hedges’ *g*) for volumetric differences across the left and right hemispheres, per region, coloured by significance (red dot/black outline/coloured in = FDR *P* < 0.05). Positive effect sizes (red) indicate larger values in individuals with chronic pain, and negative effect sizes (blue) indicate smaller values in individuals with chronic pain. Black error bars indicate 95% confidence intervals, and grey error bars indicate prediction intervals. (**B**) Same comparisons adjusted for total ICV using the residuals method. All effects are coloured for significance in the same manner as **A**. Black and grey error bars indicate 95% confidence intervals and prediction intervals, respectively.

Unadjusted volumetric reductions were detected in the left parahippocampal gyrus, right lingual gyrus, and bilaterally in the entorhinal cortex—notably, the only region to show bilateral alterations across classic structural metrics. After adjusting effect size estimates for total ICV, no regions remained significant despite minimal changes in effect size magnitude (Fig. 3A–B). There were no significant changes in surface area across the cortex.

**Figure 4:**
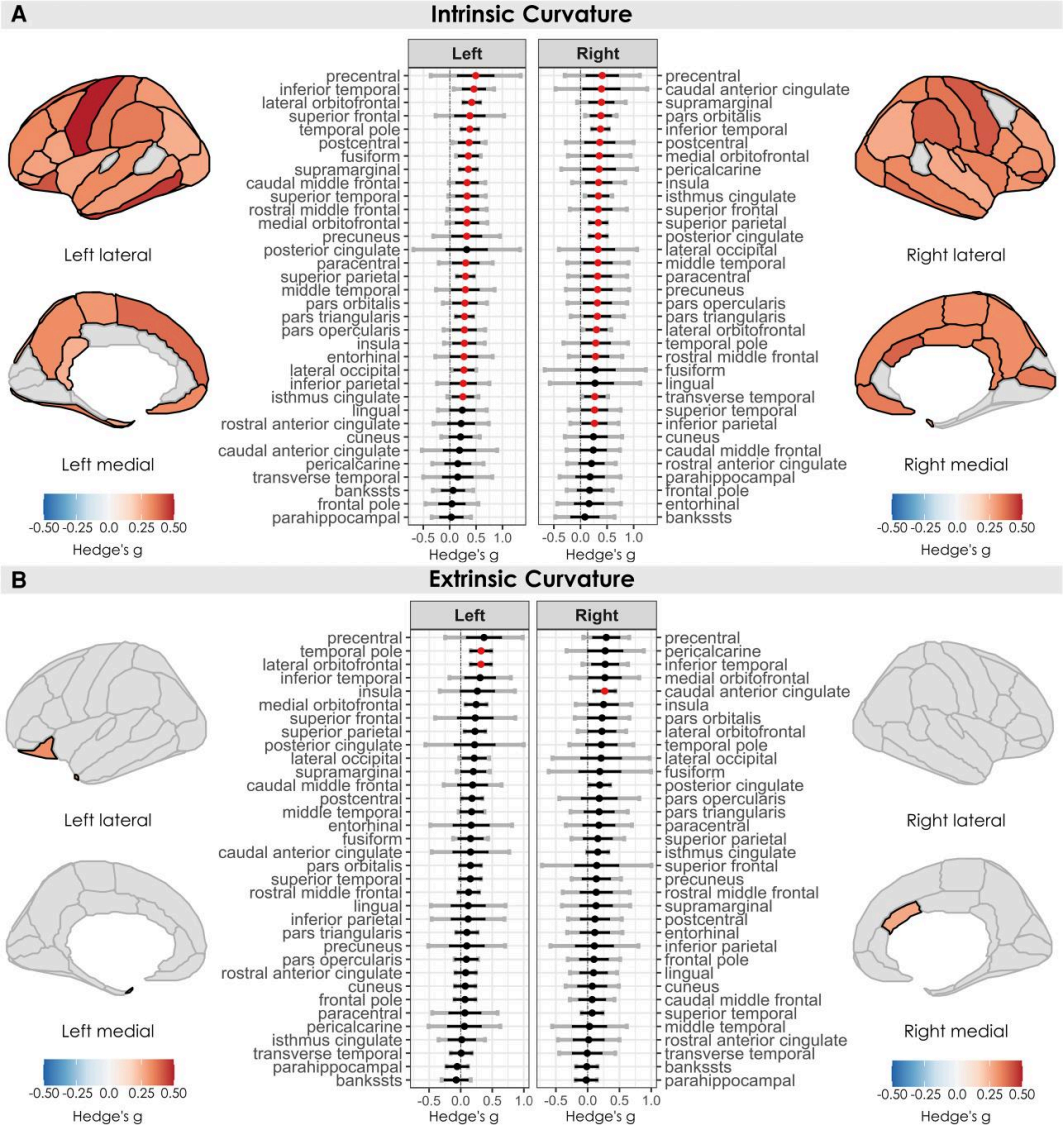
**Regional changes in intrinsic and extrinsic curvature in individuals with chronic pain.** Estimated effect sizes (Hedges’ *g*) for intrinsic (**A**) and extrinsic curvature (**B**) per region, across *k* = 8 studies, between individuals with chronic pain (*n* = 401) and healthy controls (*n* = 245), coloured by significance (red dot/black outline/coloured in = FDR *P* < 0.05). Positive effect sizes (red) indicate larger values in individuals with chronic pain, and negative effect sizes (blue) indicate smaller values in individuals with chronic pain. Black error bars indicate 95% confidence intervals, and grey error bars indicate prediction intervals.

We observed significant increases in both intrinsic and extrinsic curvature in individuals with chronic pain (Fig. 4). Intrinsic curvature effects were widespread (49 regions, 21 of which were affected bilaterally), whereas extrinsic curvature effects were restricted to the left lateral orbitofrontal cortex, the left temporal pole, and the right caudal anterior cingulate cortex. All meta-analysis statistics are provided in the supplementary table.

Overall, between-study heterogeneity was low to moderate. Significant heterogeneity (Cochrane’s Q, adjusted *P* < 0.05) was observed in seven region-metric combinations, five of which include the intrinsic curvature of the left posterior cingulate, right fusiform, right caudal anterior cingulate, right lingual cortex, and the left precentral gyrus. The remaining two were the extrinsic curvature of the superior frontal gyrus and the volume of the rostral middle frontal gyrus. Heterogeneity statistics are provided in the supplementary table.

**Figure 5:**
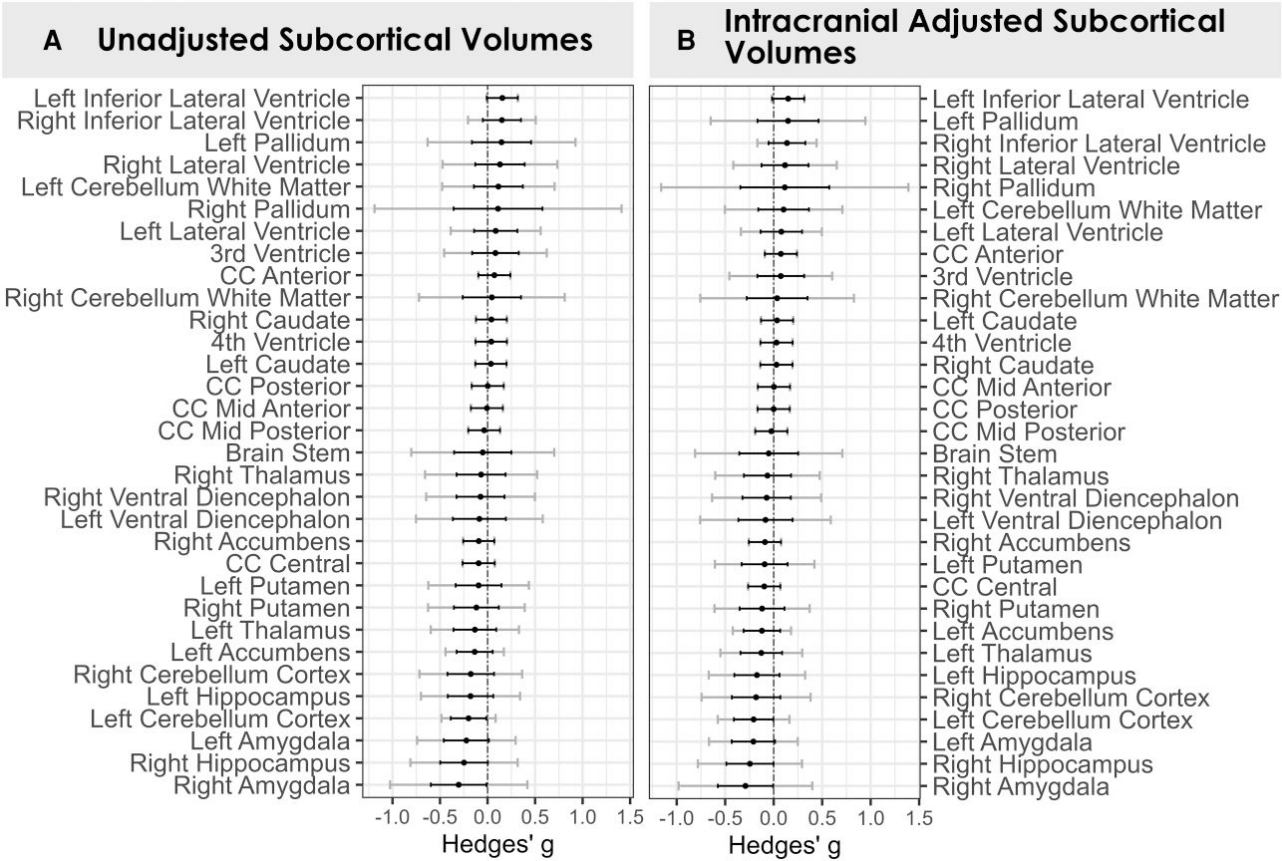
**Subcortical volumetric differences in individuals with chronic pain, before and after adjusting for total ICV.** Regional differences in grey matter volume of subcortical structures between individuals with chronic pain (*n* = 401) and healthy controls (*n* = 245) across *k* = 8 studies, coloured for significance following FDR correction (red dot = *P* < 0.05). (**A**) Unadjusted volumetric differences across subcortical structures. Positive effect sizes indicate larger volumes in individuals with chronic pain, and negative effect sizes indicate smaller volumes in individuals with chronic pain. Black error bars indicate 95% confidence intervals, and grey error bars indicate prediction intervals. (**B**) Same volumetric comparisons as **A**, adjusted for total ICV using the residuals method. Black and grey error bars indicate 95% confidence intervals and prediction intervals, respectively. Abbreviation: CC, cingulate cortex.

No significant alterations were detected in subcortical volumes in individuals with chronic pain before or after adjusting for total ICV (Fig. 5A). Adjusting for total ICV resulted in minimal changes in magnitude to effect sizes (Fig. 5B). Adjusted effect size estimates were largely negligible (|*g*| < 0.2), except for the amygdala [left, −0.209 (−0.433, 0.014); right, −0.291 (−0.576, −0.006)], hippocampus [right, −0.245 (−0.488, 0.002)], and cerebellar cortex [left, −0.207 (−0.410, 0.003)], and were generally symmetrical across the left and right hemisphere (e.g. the left and right amygdala share similar effect sizes in magnitude and directionality).

Five subcortical regions showed significant heterogeneity, with the right and left pallidum showing the widest prediction intervals [right, (−1.15, 1.39); left, (−0.65, 0.946)]. Other regions displaying significant heterogeneity were the right cerebellar white matter, left ventral diencephalon, and brain stem. All heterogeneity statistics are provided in the supplementary table.

### Sex effects

Within individual brain regions, there were no significant sex effects for any of the structural or geometric metrics. There were similarly no significant regional sex effects for subcortical volumes. Statistics for sex effects are reported in the supplementary table.

### RShiny app

We developed a publicly accessible RShiny web application that allows users to explore the raw data, study-level comparisons, and results from the IPD meta-analysis. (https://lokeryan.shinyapps.io/chronic_pain/)

## Discussion

The aim of our study was to determine changes in brain morphology associated with chronic pain independent of specific aetiologies. Importantly, pain perception arises from central nervous system processing that gates and integrates nociceptive input, and structural brain differences may therefore reflect mechanisms that contribute to, maintain, or result from chronic pain. We applied identical preprocessing pipelines and quality inspection across eight publicly available T1-weighted MRI datasets. We then performed an IPD meta-analysis of surface-based brain metrics, thereby mitigating analytic heterogeneity, and incomplete reporting biases baked into conventional aggregate data meta-analyses. For cortical thickness, surface area, and volume, our results point to a restricted number of cortical regions that were only minorly affected by chronic pain. In contrast, chronic pain was associated with widespread, nearly global increases in intrinsic curvature. All significant effects exceeded the threshold for a small effect size (|*g*| > 0.2) but remained below the threshold for a medium effect size (|*g*| < 0.5). These results provide new insights into the neuroanatomical correlates of chronic pain, highlighting the potential value of brain curvatures as a novel biomarker of disease.

### Conventional metrics: volume, surface area, and cortical thickness.

The results of our IPD analysis generally aligned with those of previously published aggregate meta-analyses examining conventional volumetric, surface area, and thickness metrics. The overall direction (i.e. reductions) and effect magnitude (i.e. small) of chronic pain on brain structure is in close agreement with a recent anatomic likelihood estimate analysis.^10^ In this recent work, significant effects were only reported after relaxing statistical thresholds. Other similarities emerged—for example, both aggregate and our IPD meta-analyses indicate that the parahippocampal gyrus—a limbic structure involved in pain modulation—has a prominent role in chronic pain.^11,45^

Volumetric reductions of the entorhinal cortex in individuals with chronic pain initially represented a bilateral morphometric change detected by way of IPD meta-analysis, not widely reported elsewhere. However, this finding did not survive adjustment for total ICV. Notably, the effect size magnitude changed minimally, suggesting the loss of significance reflects reduced statistical precision rather than absence of a biological effect. The entorhinal cortex plays a central role in episodic memory and serves as a critical hub linking the neocortex and hippocampus.^46^ Its engagement in pain-related learning and memory may underlie its vulnerability as chronic pain has been theorized as a maladaptive memory in which emotional and contextual associations persist beyond tissue healing.^17,47^ This pattern may reflect pathological remodelling driven by persistent activation, metabolic stress, or disrupted connectivity within temporal and limbic circuits. While these findings warrant further investigation in larger samples, causality cannot be inferred from cross-sectional data.

Differences emerged in comparison to aggregate data meta-analyses; however, we observed no significant associations between subcortical volumes and chronic pain before and after adjusting for total ICV. Reduced thalamus^7,11,13^ and amygdala volumes,^8^ as well as increased volumes in the caudate^7^ and cerebellum,^9^ have been reported elsewhere. Our IPD meta-analysis of subcortical volumes yielded high heterogeneity in regions like the pallidum and amygdala, which may reflect condition-specific effects. Larger, multi-condition datasets, such as those being gathered by the ENIGMA-Chronic Pain group, are needed to clarify the role of subcortical structures across and within specific chronic pain conditions.^48^ Our volumetric analysis also did not detect widespread structural changes in regions of the default mode network (DMN), commonly identified as the primary network disrupted in chronic pain functional connectivity studies.^49-51^ This dissociation between volume and functional studies suggests that chronic pain may involve network-level reorganization that exceeds detectable volumetric atrophy of these regions.

### Intrinsic and extrinsic curvature: novel morphometric biomarkers for chronic pain?

While changes in traditional morphometric measures like volume, thickness, and surface area provide valuable insights into brain structure, they represent only one aspect of structural remodelling in the brain. To our knowledge, this study is the first to incorporate curvature-based metrics to identify common structural patterns of change across multiple chronic pain conditions.

Geometric properties of the cortical surface, including intrinsic and extrinsic curvature, have emerged as promising but underutilized markers of cortical reorganization with the potential to capture more subtle changes that may be missed by conventional metrics.^22^ Extrinsic curvature reflects the surface’s shape in a three-dimensional space (e.g. a crumpled piece of paper can be flattened).^52^ Mean or extrinsic curvature corresponds with gyrification of the cortex, with sulci and gyri giving rise to different estimates.^23^ On the basis of this knowledge, increased extrinsic curvature associated with chronic pain may reflect an increase in the number of sulci ‘wrinkling’ in a given region. Our IPD meta-analysis identified a few significant associations with low inter-study heterogeneity, including the right caudal anterior cingulate—a prominent region of the brain thought to be involved in the emotional and affective dimensions of processing pain.^53^

Intrinsic curvature is a fundamental geometric property of the cortical surface that cannot be altered without distortion (e.g. a ball cannot be flattened without tearing).^52^ Gaussian (intrinsic) curvature captures finer-scale spatial variations compared to extrinsic curvature, and at the millimetre–centimetre scale, it is thought to reflect tangential organization of cortical connections, with higher curvature reflecting greater short-range projections.^23^

In chronic pain, increases in intrinsic curvature may reflect enhanced local connectivity and reduced long-range integration. For example, increased intrinsic curvature in regions like the postcentral gyrus and insula indicates reinforcement of somatosensory and salience-related circuits, contributing to sensory amplification, pain hypervigilance, and difficulty disengaging from pain.^54,55^ Such patterns parallel functional connectivity findings of strengthened local network coherence and diminished large-scale integration in chronic pain,^16,56^ suggesting a structural correlate of network segregation.

Notably, the observed global increases in intrinsic curvature span regions across multiple functional networks including the DMN, central executive network, and salience network, the same tripartite network system recently identified as the core dysfunction in chronic pain using resting state connectivity.^51^ The widespread nature of changes in intrinsic curvature—unbound by regions involved in pain processing—suggests that intrinsic curvature may capture distributed structural reorganization underlying the large-scale functional network disruptions characteristic of chronic pain. Mechanistically, differential cortical expansion producing intrinsic curvature alters neuronal spacing and shifts connectivity distributions towards more short-range connections,^23^ potentially establishing an architectural foundation that constrains network dynamics and perpetuates chronic pain even after peripheral nociceptive input resolves.

### The role of sex on brain structure in chronic pain

To our knowledge, few studies have comprehensively examined the role of sex on brain morphology in individuals with chronic pain. Among studies that have examined the effect of sex, changes in brain structure are reportedly greater in women compared to men.^57^ These studies are limited by small sample sizes, particularly with regard to the number of male participants (see Table 1 in reference),^57^ thus statistically underpowered. Based on our IPD meta-analysis, brain structure is mostly similar between men and women, insomuch as no single change in region was sexually dimorphic for any given MRI metric.

### Limitations

The main limitation of our study was that we relied entirely on publicly available T1w MRI datasets, some of which were heavily skewed towards female participants. Both Fibromyalgia datasets were comprised of only female subjects, and the migraine dataset was skewed approximately 7:1 towards females. This imbalance may confound the interpretation of sex-related effects, as datasets lacking male participants cannot contribute to sex comparisons. The large migraine dataset may also disproportionately influence the overall estimates. In addition, we were unable to access datasets that were not publicly shared; such datasets—whether withheld due to null findings, data sharing preferences, or other considerations—could not be included. Traditional publication-bias assessments are not applicable in this context because we analysed raw MRI data rather than published effect sizes or IPD. As a result, this study may be subject to a form of data-availability bias analogous to the file-drawer problem. Furthermore, we made no attempts to contact authors to access data not publicly available. The inclusion of more data would be an asset for our study and any further exploration.

Our study was limited by incomplete data on pain characteristics (severity, duration, location), comorbidities like depression, and long-term analgesic use. While Table 1 provides available medication data, obtaining complete medication histories in chronic pain patients is difficult given the frequent cycling of interventions. This is notable as some analgesics (e.g. ibuprofen^58^ and carbamazepine^59^) may alter brain morphology. Additionally, the cross-sectional design prevents causal inference, although to our knowledge, no publicly available longitudinal MRI datasets exist for chronic pain populations.

Lastly, automated segmentation using FreeSurfer recon-all is subject to known limitations, including potential regional over- or underestimation.^60,61^ These were mitigated through automated outlier detection and targeted visual inspection. However, due to the scale of the dataset, visual review was necessarily selective rather than exhaustive, and subtle segmentation inaccuracies may persist.

## Conclusion

We performed an IPD meta-analysis examining structural changes in the brain in individuals with diverse chronic pain conditions. Our goal was to elucidate a structural signature of chronic pain. Our approach revealed widespread cortical alterations, including bilateral volumetric reductions in the entorhinal cortex and global increases in intrinsic curvature. These findings support the view of chronic pain as a disorder of large-scale cortical reorganization, extending beyond sensory regions. Notably, the structural patterns identified in our analysis align with functional imaging reports of strengthened local network connectivity and reduced long-range integration, converging on the concept of chronic pain as a disconnected network disease state of the brain. By applying uniform processing and statistical models across datasets, this study offers a standardized framework that can be built upon in future work. Expanding this approach to larger, more diverse cohorts, including longitudinal datasets, will help delineate condition-specific effects and address causal relationships in brain reorganization.

## Supplementary Material

fcag146_Supplementary_Data

## Data Availability

Beyond current analyses, we developed an RShiny application (https://lokeryan.shinyapps.io/chronic_pain/) that enables interactive visualization of the raw data, study-level comparisons, and meta-analyses. This tool can allow other researchers to explore analyses in detail and facilitate new lines of inquiry beyond the scope of the present study. All MRI images analysed for this study were obtained from OpenNeuro (https://openneuro.org/), an open-science neuroinformatics database, or OpenPain (https://openpain.org/), an open access data sharing platform for brain imaging studies of human pain. All study-level and sex analysis results can be found in the Supplementary material and can be explored in the RShiny application. In-house bash scripts used to process the MRI images have been uploaded to our GitHub repository, along with the generated R scripts used to analyse all datasets. Additionally, code generated for the purpose of data processing and statistical analysis is included in the following GitHub: https://github.com/lokeryan/ChronicPainIPD. Upon reasonable request, the corresponding author can provide additional information and data to interested researchers for the purpose of academic research and further scientific investigations. If interested, other investigators may contribute their data to our study by reaching out to the corresponding author, which we can implement into our RShiny application. All study-level and meta-analysis results can be found in the supplementary. Please refer to the supplementary table dictionary for descriptions of all sheet names and column variables.
