# Supplementary material for: Convergent structural brain alterations in chronic pain: a multi-metric individual participant data meta-analysis
Source: Brain Commun. 2026 Apr 24;8(3):fcag146. doi: 10.1093/braincomms/fcag146 (PMC13148768; doi:10.1093/braincomms/fcag146)
Supplement: fcag146_Supplementary_Data [file fcag146_supplementary_data.zip › Supplementary_Table_Dictionary.docx]

**Supplementary Tables Breakdown – 16 Total sheets**

**General Abbreviations**

| study | Meaning/study |
| --- | --- |
| OA | Osteoarthritis: Tetreault, 2016,^1^ |
| FM1 | Fibromyalgia: Pando-Naude, 2019,^2^ |
| FM2 | Fibromyalgia: Balducci, 2022,^3^ |
| CLBP1 | Chronic Low Back Pain: Makary, 2020,^4^ |
| CLBP2_S1 | Chronic Low Back Pain: Mano, 2018 (UK Data),^5^ |
| CLBP3_S2 | Chronic Low Back Pain: Mano, 2018 (Japan Data),^5^ |
| migraine | Migraine: Seminowicz, 2020,^6^ |
| ptn | Primary Trigeminal Neuralgia: Filimonova, 2025,^7^ |

**Measurement Types**

| Abbreviation | Definition |
| --- | --- |
| SurfArea | Surface Area (mm^2^) |
| GrayVol | Grey Matter Volume (mm^3^) |
| ThickAvg | Average Cortical Thickness (mm) |
| MeanCurv | Mean Curvature – extrinsic curvature (1/mm) |
| GausCurv | Gaussian Curvature – intrinsic curvature (1/mm^2^) |

**Sheets 1-6: Study level results**

**S_Cort –** study level cortical results, includes unadjusted intracranial volume (ICV) volumetric comparisons

**ICV_S –** ICV adjusted study level cortical results for grey matter volume.

**S_Cort_Sex** – study level cortical results, stratified by sex, includes unadjusted ICV volumetric comparisons.

**S_Subcort –** Unadjusted ICV volumetric analysis of subcortical structures at the study level.

**ICV_S_Subcort –** ICV adjusted subcortical volumetric comparison, at the study level.

**S_Subcort_Sex –** study level subcortical results, stratified by sex, not adjusted for ICV

General Column Names

| Column Name | Meaning |
| --- | --- |
| FDR_p | p-value corrected for multiple comparisons, grouped by measurement metric |
| Mean_Group1 | Average value per measurement of HEALTHY CONTROLS |
| SD_Group1 | Standard deviation of healthy controls data |
| N_Group1 | Sample size of healthy controls per dataset |
| Mean_Group2 | Average value per measurement of INDIVIDUALS WITH CHRONIC PAIN |
| SD_Group2 | Standard deviation of individuals with chronic pain data |
| N_Group2 | Sample size of individuals with chronic pain per dataset |
| Hedges_g | Effect size (hedge’s g) – positive indicates larger value in individuals with chronic pain, and negative value indicates smaller values in individuals with chronic pain. |
| SE_Hedges_g | Standard error of hedge’s g |
| CI_lower | Lower confidence interval of Hedge’s G |
| CI_upper | Upper confidence interval of Hedge’s G |

**Meta Analysis Results**

**Sheets 7-16**

**M_Cort –** Meta-analysis results of cortical analyses, includes unadjusted ICV volumetric comparisons

**ICV_M –** Meta-analysis results of ICV adjusted cortical volumes

**ICV_M_Sex –** Sex analysis of ICV adjusted cortical volumes, of meta-estimates

**M_Cort_Sex** – Meta results of sex stratified, study-level cortical comparisons, includes unadjusted ICV volumetric comparisons.

**Cort_Sex_Analysis –** Sex analysis of cortical level meta-analyses, includes unadjusted ICV comparisons

**M_Subcort –** Meta-analysis results of subcortical analyses, includes unadjusted ICV comparisons

**ICV_M_Subcort –** ICV adjusted, meta-analysis results of subcortical analysis**.**

**M_Subcort_Sex –** Meta results of sex stratified, study-level unadjusted subcortical comparisons

**Subcort_Sex_Anaylsis –** Sex analysis of unadjusted subcortical level meta-analyses**.**

**ICV_M_Subcort_Sex –** Sex analysis of subcortical level meta-analysis of adjusted volumes**.**

| Column Names | Meaning |
| --- | --- |
| K | Number of studies included in analysis |
| Estimate | Estimated pooled effect size of given region/measurement. Positive indicates a larger value in individuals with chronic pain, and negative values indicate a smaller value in individuals with chronic pain. |
| SE | Standard error of estimate |
| Zval | Z statistic of pooled effect size |
| Pval | Probability value for significance test of estimated effect size |
| FDR | P-value corrected for multiple comparisons, grouped by metric. |
| CI_lb | Confidence interval, lower bound of estimated effect size |
| CI_ub | Confidence interval, upper bound of estimated effect size |
| PI_lb | Prediction interval, lower bound, of estimated effect size |
| PI_ub | Prediction interval, upper bound, of estimated effect size |
| I2 | $I^{2}$, percentage of total variation across studies due to heterogeneity |
| T2 | $\tau^{2}$, random-effects variance component for between study heterogeneity |
| Q | Cochran’s Q statistic for heterogeneity |
| Pval_Q | p-value for Cochran’s Q test of heterogeneity |
| FDR_Q | Cochran’s Q test p-value corrected for multiple comparisons |
| Z_diff | z-score calculated from Wald-z test |
| P_z | P-value for Wald-Z test |
| FDR_p_z | Wald-z test p-value corrected for multiple comparisons, grouped by measurement. |

**NOTE:** for analysis sheets, column names ending with “_m” indicate male specific outcomes, and column names ending with “_f” indicate female specific outcomes. E.g., estimate_f indicates estimated pooled effect size of a given region & measurement for females. Some columns may also not contain renamed hemispheres, lh refers to left hemisphere and rh refers to right hemisphere.

**References**

1. Tétreault P, Mansour A, Vachon-Presseau E, Schnitzer TJ, Apkarian AV, Baliki MN. Brain Connectivity Predicts Placebo Response across Chronic Pain Clinical Trials. *PLoS Biol*. 2016;14(10):e1002570. doi:10.1371/journal.pbio.1002570

2. Pando-Naude V, Barrios FA, Alcauter S, et al. Functional connectivity of music-induced analgesia in fibromyalgia. *Sci Rep*. 2019;9(1):15486. doi:10.1038/s41598-019-51990-4

3. Balducci T, Rasgado-Toledo J, Valencia A, van Tol MJ, Aleman A, Garza-Villarreal EA. A behavioral and brain imaging dataset with focus on emotion regulation of women with fibromyalgia. *Sci Data*. 2022;9(1):581. doi:10.1038/s41597-022-01677-9

4. Makary MM, Polosecki P, Cecchi GA, et al. Loss of nucleus accumbens low-frequency fluctuations is a signature of chronic pain. *Proc Natl Acad Sci U S A*. 2020;117(18):10015-10023. doi:10.1073/pnas.1918682117

5. Mano H, Kotecha G, Leibnitz K, et al. Classification and characterisation of brain network changes in chronic back pain: A multicenter study. *Wellcome Open Res*. 2018;3:19. doi:10.12688/wellcomeopenres.14069.2

6. Seminowicz DA, Burrowes SAB, Kearson A, et al. Enhanced mindfulness-based stress reduction in episodic migraine: a randomized clinical trial with magnetic resonance imaging outcomes. *Pain*. 2020;161(8):1837-1846. doi:10.1097/j.pain.0000000000001860

7. Filimonova E, Pashkov A, Moysak G, Martirosyan A, Rzaev J. Hippocampal Subfield Abnormalities in Patients With Primary Trigeminal Neuralgia. *Journal of Neuroimaging*. 2025;35(1). doi:10.1111/jon.70026
